# Clinicopathological characteristics of patients with amyotrophic lateral sclerosis resulting in a totally locked-in state (communication Stage V)

**DOI:** 10.1186/s40478-016-0379-3

**Published:** 2016-09-30

**Authors:** Kentaro Hayashi, Yoko Mochizuki, Ryoko Takeuchi, Toshio Shimizu, Masahiro Nagao, Kazuhiko Watabe, Nobutaka Arai, Kiyomitsu Oyanagi, Osamu Onodera, Masaharu Hayashi, Hitoshi Takahashi, Akiyoshi Kakita, Eiji Isozaki

**Affiliations:** 1 Department of Neurology, Tokyo Metropolitan Neurological Hospital, 2-6-1 Musashidai, Fuchu-shi, Tokyo 183-0042 Japan; 2 Department of Pathology, Tokyo Metropolitan Neurological Hospital, 2-6-1 Musashidai, Fuchu-shi, Tokyo 183-0042 Japan; 3 Department of Neurology, Tokyo Metropolitan Kita Medical and Rehabilitation Center for the Disabled, 1-2-3 Jujodai, Kita-ku, Tokyo 114-0033 Japan; 4 Department of Neurology, Brain Research Institute, Niigata University, 1-757 Asahimachidori, Chuo-ku, Niigata 951-8585 Japan; 5 Department of Pathology, Brain Research Institute, Niigata University, 1-757 Asahimachidori, Chuo-ku, Niigata-shi, Niigata 951-8585 Japan; 6 Department of Sensory and Motor System, Tokyo Metropolitan Institute of Medical Science, 2-1-6 Kamikitazawa, Setagaya-ku, Tokyo 156-8506 Japan; 7 Department of Laboratory Neuropathology, Tokyo Metropolitan Institute of Medical Science, 2-1-6 Kamikitazawa, Setagaya-ku, Tokyo 156-8506 Japan; 8 Division of Neuropathology, Department of Brain Disease Research, Shinshu University School of Medicine, 3-1-1 Asahi, Matsumoto-shi, Nagano 390-8621 Japan; 9 Department of Brain Development and Neural Regeneration, Tokyo Metropolitan Institute of Medical Science, 2-1-6 Kamikitazawa, Setagaya-ku, Tokyo 156-8506 Japan

**Keywords:** Amyotrophic lateral sclerosis, Communication Stage V, Totally locked-in state, Pallido-nigro-luysian system, Brainstem reticular formation, Cerebral cortical degeneration

## Abstract

In the present study, we performed a comprehensive analysis to clarify the clinicopathological characteristics of patients with amyotrophic lateral sclerosis (ALS) that had progressed to result in a totally locked-in state (communication Stage V), in which all voluntary movements are lost and communication is impossible. In 11 patients, six had phosphorylated TAR DNA-binding protein 43 (pTDP-43)-immunoreactive (ir) neuronal cytoplasmic inclusions (NCI), two had fused in sarcoma (FUS)-ir NCI, and three had copper/zinc superoxide dismutase (SOD1)-ir NCI. The time from ALS onset to the need for tracheostomy invasive ventilation was less than 24 months in ten patients. Regardless of accumulated protein, all the patients showed common lesions in the pallido–nigro–luysian system, brainstem reticular formation, and cerebellar efferent system, in addition to motor neurons. In patients with pTDP-43-ir NCI, patients with NCI in the hippocampal dentate granule neurons (DG) showed a neuronal loss in the cerebral cortex, and patients without NCI in DG showed a preserved cerebral cortex. By contrast, in patients with FUS-ir NCI, patients with NCI in DG showed a preserved cerebral cortex and patients without NCI in DG showed marked cerebral degeneration. The cerebral cortex of patients with SOD1-ir NCI was preserved. Together, these findings suggest that lesions of the cerebrum are probably not necessary for progression to Stage V. In conclusion, patients with ALS that had progressed to result in communication Stage V showed rapidly-progressed symptoms, and their common lesions could cause the manifestations of communication Stage V.

## Introduction

Patients with amyotrophic lateral sclerosis (ALS) dependent on tracheostomy with invasive ventilation (TIV) use mostly nonverbal communication and find it difficult to communicate as their muscle weakness progresses. In a previous study [9], we proposed a classification system for the communication abilities of patients with advanced ALS that consists of five stages: Stage I, communicates in sentences; Stage II, communicates with one-word answers only; Stage III, communicates with nonverbal yes/no responses only; Stage IV, occasionally cannot communicate due to uncertain yes/no responses; and Stage V, cannot communicate by any means. We also analyzed the relationship between clinical findings and the prognosis for communication disturbance [9, 19]. At present, communication Stage V is indicative of a “totally locked-in state” [7, 8]. Our previous analysis of 29 autopsies of patients with ALS who were dependent on TIV showed that seven patients who progressed to Stage V had begun to require TIV significantly earlier than patients who died in Stage IV or earlier, and the patients who progressed to Stage V frequently had a family history of ALS and gene mutation [9]. Further study showed that need for TIV, impaired oculomotor movement, and becoming totally quadriplegic within 24 months of ALS onset were predictors of severe communication impairment. Therefore, we recommended early detection of impaired communication and identification of the best methods of communication [19]. The first neuropathological reports of two patients with ALS who progressed to Stage V [7] showed severe multisystem degeneration. By contrast, Oyanagi et al. [22] reported marked preservation of the visual and olfactory pathways in patients with ALS who progressed to Stage V. There are reports of patients with lesions in their primary motor cortex, but a preserved cerebral cortex [10, 14, 16, 24]. In contrast, patients with marked cerebral atrophy due to degeneration of the cerebral cortex and white matter have also been reported [11, 17, 20]. Neuroradiologically, a progressive cerebral atrophy has been shown in siblings with ALS who carried a mutation in the gene for optineurin (*OPTN*) [28]. However, the distribution and characteristics of the cerebral lesions are unclear. To date, clinicopathology of patients with ALS who progressed to communication Stage V has been reported only in patient reports [7, 10, 11, 14, 16, 17, 20, 24, 26, 27]. Therefore, in the present study we performed a comprehensive analysis of the clinicopathological features and immunohistochemical characterization of patients with ALS who had progressed to communication Stage V.

## Materials and methods

### Patients

By examining medical records, we enrolled 11 (3.4 % of studied patients with ALS neuropathology) Japanese patients with ALS who had progressed to Stage V (Table 1), from among 320 patients with ALS neuropathologically confirmed at autopsy at the Tokyo Metropolitan Neurological Hospital between 1980 and 2012 (150 patients), and in the Department of Pathology Brain Research Institute, Niigata University between 1963 and 2012 (170 patients). No patients had clinical manifestations of either cognitive or behavioral impairment before progressing to Stage V. In addition, no patients showed clinical evidence of anoxia, such as suffocation, artificial ventilator accident, or blood pressure decrease to <80 mmHg with shock, during their clinical course. Several patients were reported elsewhere as having ALS that had progressed to Stage V [7, 10, 16, 17, 20, 24]; however, some of the older reports did not provide immunohistochemical characterization of patient specimens. The clinicopathological features of patients 4 and 11 were previously reported as motor neuron disease [27] and we reevaluated these patients as having phosphorylated TAR DNA-binding protein 43 (pTDP-43)-immunoreactive (ir) neuronal cytoplasmic inclusions (NCI) and copper/zinc superoxide dismutase (SOD1)-ir NCI respectively. Therefore, we included them in the present study, and reevaluated the clinicopathological and immunohistochemical features of all 11 patients using the same criteria. Of the 11 patients, six (patients 1–6) had pTDP-43-ir NCI, two (patients 7, 8) had fused in sarcoma (FUS)-ir NCI, and three (patients 9–11) had SOD1-ir NCI (Table 1). This study was approved by the Ethical Review Boards of Tokyo Metropolitan Neurological Hospital and Niigata University.

**Table 1:** Clinical characteristics

					Time from onset to clinical event (months)						
Patients	Sex	Age at onset(years)	Disease duration(months)	Disease duration(months)	Need for tracheostomy invasive ventilation	Progression to total quadriplegia	Development of overt oculomotor limitation	Resulting in communication Stage V^a^	Time from Stage V to death(months)	Accumulated protein	Reference
1	F	62	104	LE	12	34	33	37	67	TDP-43	[20]
2	F	52	161	UE	12	25	14	76	85	TDP-43	
3	F	53	120	B	56	120	120	120	0	TDP-43	
4	M	73	78	B	5	na	11	29	49	TDP-43	[27] patient 2
5	M	60	117	UE	15	31	18	81	36	TDP-43	[7] case 1
6	M	64	57	UE	12	33	33	56	1	TDP-43	[7] case 2
7	F	13	312	LE	18	18	36	120	192	FUS	[17] patient 1
8	M	39	102	LE	24	66	86	102	0	FUS	[16]
9	F	38	106	LE	8	9	11	36	70	SOD1	[24]
10	M	57	128	B	21	45	52	84	44	SOD1	[10]
11	M	61	29	UE	5	6	19	28	1	SOD1	[27] patient 1
mean		52.0	119.5		17.0	38.7	39.4	69.9	47.8		

*F* female, *M* male, *na* not available, *TDP-43* TAR DNA-binding protein 43, *FUS* fused in sarcoma, *SOD1* copper/zinc superoxide dismutase 1, *LE* lower extremity, *UE* upper extremity, *B* bulbar

^a^Communication Stage V is indicative of a “totally locked-in state” [7, 8], in which all voluntary movements are lost and communication is impossible by any means

### Histopathology

Specimens from the brain and spinal cord were fixed with 20 % buffered formalin and embedded in paraffin wax. Loss of neurons or fibers, or both, and gliosis were assessed in various regions of the nervous system using 10 or 4 μm sections in hematoxylin and eosin and Klüver–Barrera stains. When necessary, Bodian and Holzer stains were additionally used.

For immunohistochemistry, 6 or 4 μm sections were prepared. Sections from the frontal lobe, temporal lobe, hippocampus, parietal lobe, occipital lobe, basal ganglia, thalamus, cerebellum, midbrain, pons, medulla oblongata, and spinal cord were immunostained for pTDP-43, using a rabbit polyclonal antibody against pTDP-43 (pS409/410; CosmoBio, Tokyo, Japan) at a dilution of 1:5000 or 1:8000, FUS, using a rabbit polyclonal antibody against FUS (Sigma-Aldrich St. Louis, MO, USA) at a dilution of 1: 2000, SOD1, using a rabbit polyclonal antibody against SOD1 (Proteintech, Tokyo, Japan) at a dilution of 1:100. Required sections were immunostained for phosphorylated tau protein using the mouse monoclonal antibody AT8 (Innogenetics, Ghent, Belgium) at a dilution of 1:200, α-synuclein using a mouse monoclonal antibody against α-synuclein (Wako, Osaka, Japan) at a dilution of 1:8000 or 1:10,000, and ubiquitin using a rabbit polyclonal antibody against ubiquitin (Dako, Glostrup, Denmark) at a dilution of 1:800. Before antibody incubation, sections were treated by microwaving in citrate-buffered saline (pH 6.0, 15 min) to unmask antigens. Antibody binding was visualized using a labeled streptavidin–biotin immunoperoxidase method. The chromogen and counterstain were diaminobenzidine and hematoxylin, respectively. NCI in each patient was assessed by immunostaining with pTDP-43, FUS, or SOD1.

We evaluated the degree and extent of the neuronal loss, gliosis, and NCI semiquantitatively. Degeneration was assessed as the degree of neuronal loss and gliosis and was indicated as absent (–), slight (+) (as shown in Fig. 3i), mild (++) (as shown in Fig. 3e-g), or severe (+++) (as shown in Fig. 3a-d, h). The frequency of NCI, evaluated at 200X magnification, was indicated as follows: none (0), no NCI across the entire section; rare (1), an average of <2 NCI per 5 fields; occasional (2), an average of 2–10 NCI per 5 fields; and frequent (3), an average of >10 NCI per 5 fields. For the semiquantitative analysis, two neuropathologists (K.H. and Y.M.) observed the specimens, and their scores were almost coincident.

## Results

### Clinical characteristics 

Except for patient 7 with FUS-ir NCI, all patients had adult onset ALS (Table 1). The time from ALS onset to need for TIV was less than 24 months in ten patients, and 56 months in patient 3 with pTDP-43-ir NCI. All the patients progressed to have total quadriplegia and developed overt oculomotor limitation after using TIV. Development of oculomotor limitation started before or after they developed total quadriplegia, and the patients developed vertical gaze palsy followed by horizontal gaze palsy. Subsequently the patients showed slow eye movement and ultimately became ophthalmoplegic. All the patients used only eye movement for communication just before progressing to Stage V.

### Macroscopic findings

**Fig. 1:**
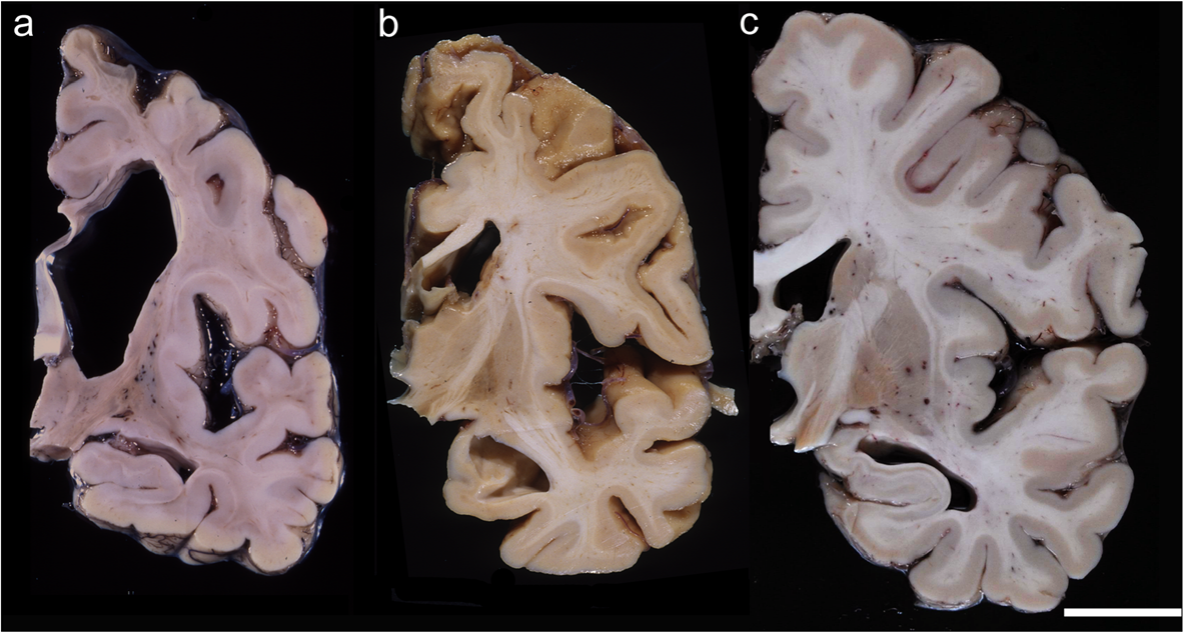
Coronal sections of the cerebrum at the subthalamic nucleus. **a** Patient 7 with fused in sarcoma (FUS)-immunoreactive (ir) neuronal cytoplasmic inclusions (NCI) showed enlargement of the anterior horn of the lateral ventricle, and atrophy of the caudate nucleus, putamen, and frontal lobe, particularly in the frontal white matter along with a thin corpus callosum. However, the hippocampus and temporal lobe were preserved. **b** Patient 2 with phosphorylated TAR DNA-binding protein 43 (pTDP-43)-ir NCI showed temporal predominately frontotemporal atrophy. **c** Patient 4 with pTDP-43-ir NCI showed no white matter atrophy nor enlargement of the lateral ventricle. The globus pallidus and subthalamic nucleus were brownish with mild to severe atrophy, while the caudate nucleus and the putamen were relatively preserved in all patients except for patient 7 (**a**), and the optic nerve was preserved in all patients. (Bar = 2 cm)

Brain weight ranged from 610 to 1395 g (mean 1032.3 g) (Table 2) before formalin fixation. Four patients (patients 1, 2 (Fig. 1b), and 3 with pTDP-43-ir NCI, and 7 with FUS-ir NCI (Fig. 1a)) showed severe cerebral atrophy, which was a brain weight of <1000 g. They showed marked atrophy of both the pons and midbrain. The brain weight of seven patients (patients 4 (Fig. 1c), 5 and 6 with pTDP-43-ir NCI, 8 with FUS-ir NCI, and 9–11 with SOD1-ir NCI) was >1000 g, and their pons and midbrain showed mild to moderate atrophy. All patients showed severe atrophy of the spinal cord and the medulla oblongata. The optic nerve (Fig. 1) and the lateral geniculate body of all the patients appeared preserved.

**Table 2:**
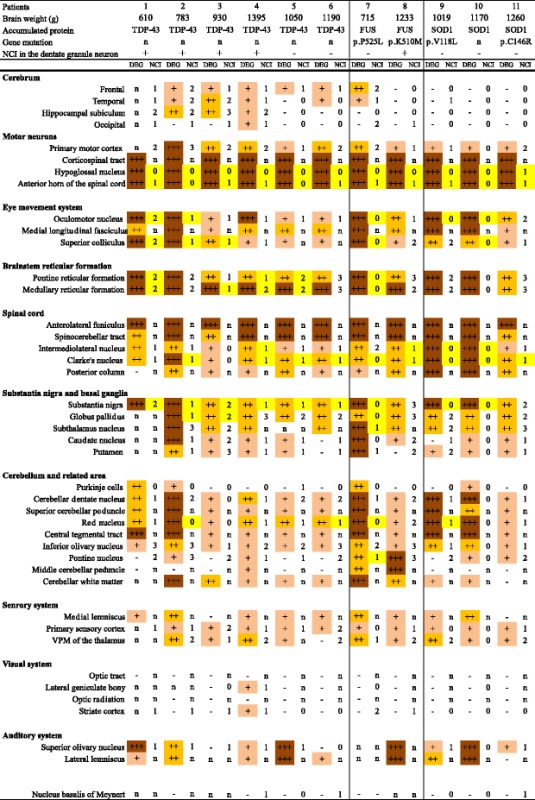
Neuropathological findings

*DEG* degeneration assessed degree of neuronal loss and gliosis on the hematoxylin and eosin, and Klüver--Barrera-stained sections

The degeneration was indicated as absent (–); slight (+); mild (++); or severe (+++)

*NCI* neuronal cytoplasmic inclusions, The NCI was indicated as none (0); rare (1); occasional (2); or frequent (3), yellow means that we were not able to evaluate the exact occurrence of NCI in the lesions, because of moderate to severe neuronal loss

*n* not evaluated (or not examined), *VPM* ventral posterior medial nucleus

### Microscopic findings

Table 2 shows the neuropathological findings of the patients.

#### Lesions of the motor system

**Fig. 2:**
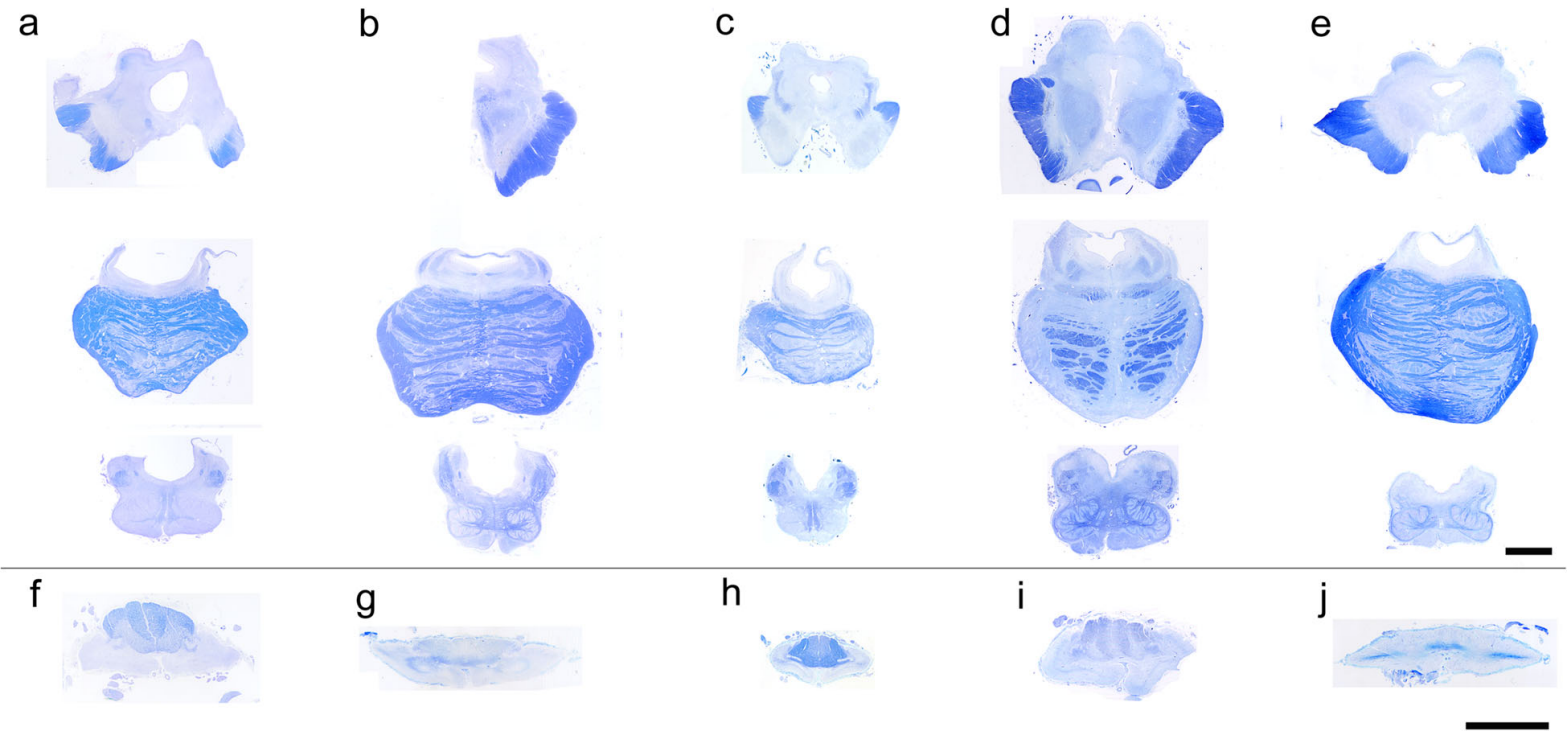
Brainstem and seventh cervical cord at the same magnification respectively. All patients showed marked nerve fiber loss of the brainstem tegmentum including the brainstem reticular formation and both lateral and anterior funiculus in addition to pyramidal tract, and showed marked atrophy of the anterior horn of the spinal cord. The medulla oblongata and spinal cords showed severe atrophy and the fourth ventricle was markedly dilatated with severe degeneration of the hypoglossal nucleus in all patients. The midbrain and pons showed severe atrophy in patients 2 with phosphorylated TAR DNA-binding protein 43 (pTDP-43)-immunoreactive (ir) neuronal cytoplasmic inclusions (NCI) (**a**) and patient 7 with fused in sarcoma (FUS)-ir NCI (**c**) in whom brain weight was less than 1000 g, mild to moderate atrophy in patient 5 with pTDP-43-ir NCI (**b**), patient 8 with FUS-ir NCI (**d**), and patient 10 with copper/zinc superoxide dismutase (SOD1)-ir NCI (**e**) in whom brain weight was more than 1000 g. The superior cerebellar peduncle in patients 5 (**b**) and 8 (**d**) were degenerated mildly. Loss of transverse fibers of the pons and middle cerebellar peduncle were observed only in patient 8 (**d**). The posterior column of the spinal cord showed a marked loss of fibers in patients 5 (**g**) and 10 (**j**), but they were relatively preserved in patient 7 (**h**), and fiber loss of the middle root zone in patient 2 (**f**) and 8 (**i**). **a**, **f** Patient 2 with pTDP-43-ir NCI. **b**, **g** Patient 5 with pTDP-43-ir NCI. **c**, **h** Patient 7 with FUS-ir NCI. **d**, **i** Patient 8 with FUS-ir NCI. **e**, **j** Patient 10 with SOD1-ir NCI, (Bar = 5 mm, Klüver–Barrera staining)

All the patients showed a severe loss of Betz cells. The other neurons in the motor cortex were mildly decreased with a few NCI in the patients with mild cerebral atrophy (patients 4–6, and 8–11). By contrast, the patients with severe cerebral atrophy (patients 1–3, and 7) showed severe neuronal loss with gliosis and many NCI in layer 2 and the deeper layer of the motor cortex. Basophilic inclusions were frequently observed in patient 7 and rarely observed in patient 8. The pyramidal tract showed severe fiber loss, although a few small fibers remained in the medullary pyramid and both the lateral and anterior corticospinal tract in the spinal cord. The anterior horn of the spinal cord and the tegmentum of the brainstem including the hypoglossal nucleus showed severe atrophy (Fig. 2) and severe neuronal loss with a few NCI in the atrophied neurons. The facial and the trochlear nuclei, which were confirmed in patients 3 and 11 showed severe neuronal loss with gliosis. The oculomotor nucleus showed a difference in the degree of degeneration. We were not able to evaluate the exact occurrence of NCI in the lesions, because of severe neuronal loss.

#### Lesions of the extrapyramidal motor system and nonmotor system

**Fig. 3:**
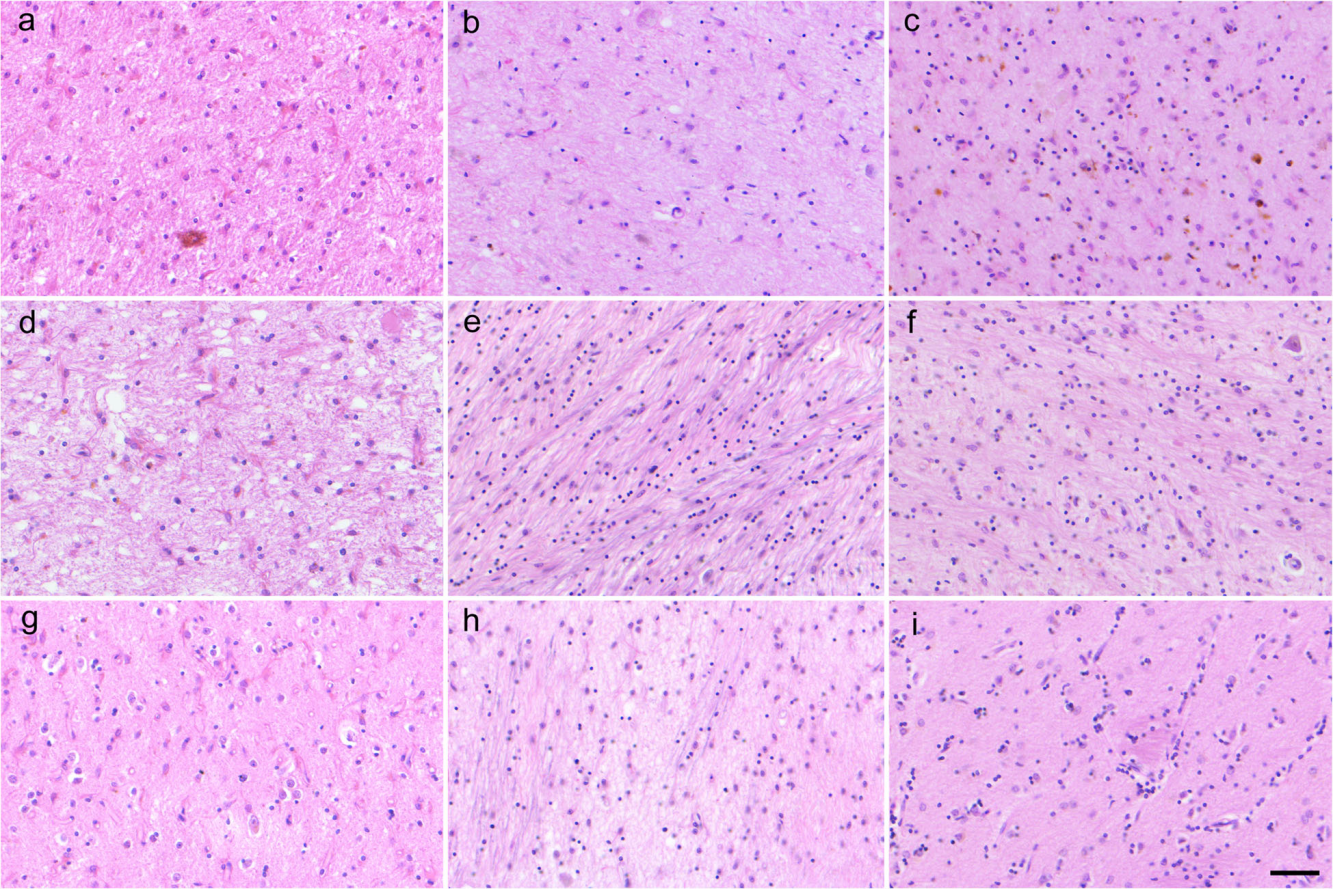
Lesions of the nonmotor system. **a**–**c** Substantia nigra showing severe neuronal loss with gliosis. **d**–**f** Globus pallidus, **g**–**i** putamen. Only patient 7 showed more severe degeneration in the putamen (**h**) than in the globus pallidus (**e**). Other patients showed more severe degeneration in the globus pallidus (**d**, **f**) than in the putamen (**g**, **i**). **a**-**d**, **h** neuronal loss and gliosis were severe (+++). **e**-**g** neuronal loss and gliosis were mild (++). **i** neuronal loss and gliosis were slight (+). **a**, **d**, **g** Patient 2 with phosphorylated TAR DNA-binding protein 43-immunoreactive (ir) neuronal cytoplasmic inclusions (NCI). **b**, **e**, **h** Patient 7 with fused in sarcoma-ir NCI. **c**, **f**, **i** Patient 10 with copper/zinc superoxide dismutase-ir NCI, (Bar = 50 μm, hematoxylin and eosin staining)

Neuronal and fiber loss with gliosis were observed in the substantia nigra (Fig. 3a–c), globus pallidus (Fig. 3d–f), subthalamic nucleus, brainstem reticular formation, cerebellar dentate nucleus, superior cerebellar peduncle, red nucleus, Clarke’s nucleus, and posterior spinocerebellar tract in all the patients. In particular, the degeneration of the substantia nigra (Fig. 3a–c), globus pallidus (Fig. 3d–f), subthalamic nucleus, and brainstem reticular formation (Fig. 2) was moderate or severe in all patients regardless of the time for progression from Stage V to death, or the type of accumulated proteins. By contrast, the cerebellar efferent system, which consists of the cerebellar dentate nucleus, superior cerebellar peduncle, and red nucleus, showed mild to moderate degeneration in patients 3, 5, and 6 with pTDP-43-ir NCI, in patient 8 with FUS-ir NCI, and in patient 11 with SOD1-ir NCI. The time for progression from Stage V to death was shorter in these five patients (0–36 months) than in the other six patients (44–192 months). The cerebellar afferent system, which consists of the pontine nuclei and middle cerebellar peduncle, was relatively preserved. However, in patient 8, who had a p.K510M mutation in the gene for FUS (*FUS*) [16], showed severe degeneration in the cerebellar afferent pathway. Patient 7, who had a p.P525L mutation in *FUS* and severe frontal lobe atrophy [17], showed severe degeneration in the caudate nucleus and putamen (Fig. 3h) and moderate degeneration in the globus pallidus (Fig. 3e). The caudate nucleus and putamen (Fig. 3g, i) were relatively better preserved than the globus pallidus (Fig. 3d, f), except for in patient 7. The inferior olivary nucleus in patients 2 and 10 showed vacuolar degeneration in neurons and an increase of gemistocytic astrocytes, indicating inferior olivary hypertrophy [5, 10]. For pTDP-43, FUS, and SOD1, no patients exhibited accumulation of multiple different proteins. AT8 immunostaining of neurofibrillary tangles revealed a Braak stage of ≤ II [1]. No patients had α-synuclein-ir structures. In addition, among patients with pTDP-43-ir NCI, no patients showed displayed ubiquitin-ir NCI in cerebellar granule cells or hippocampal CA4 subfield neurons.

#### Preserved areas

**Fig. 4:**
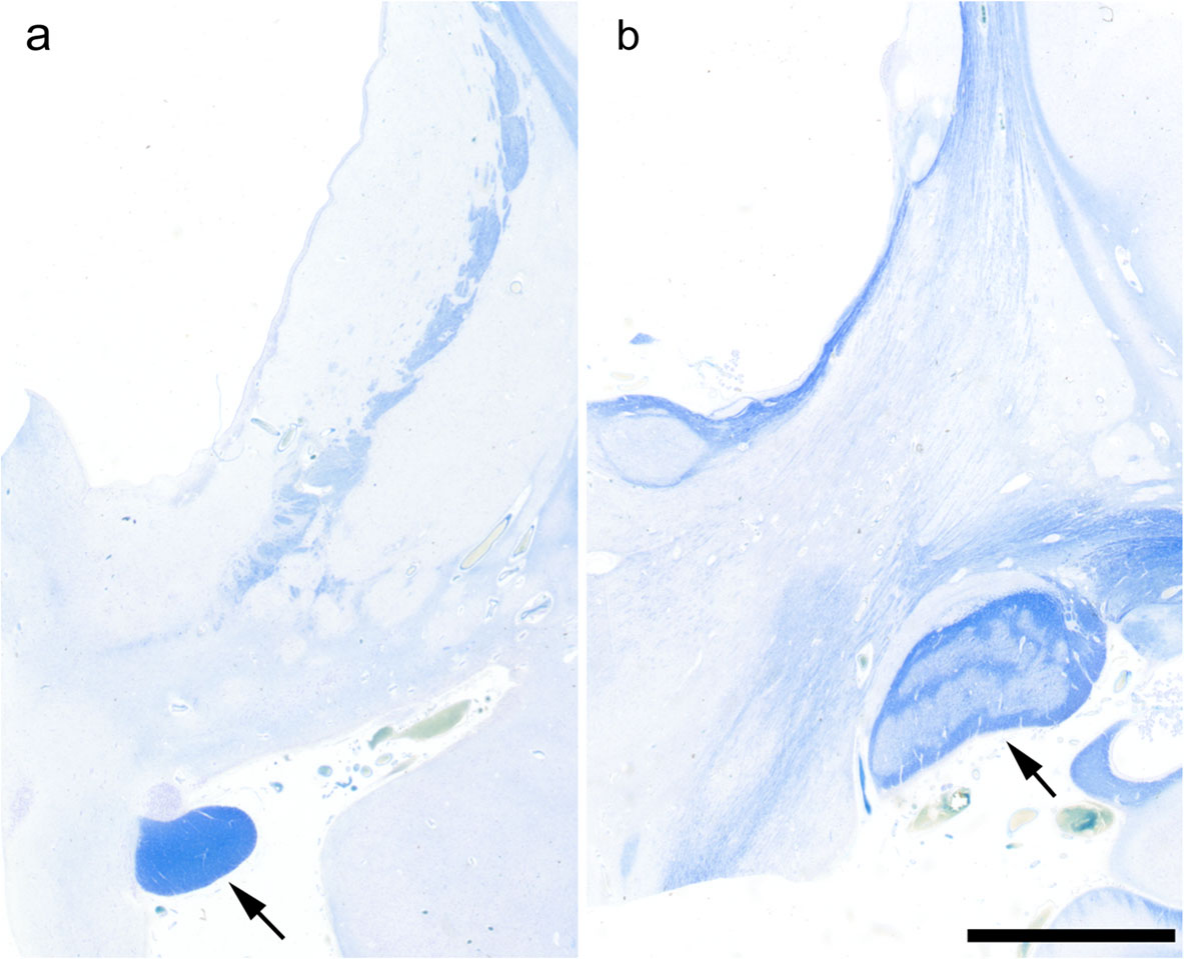
Preserved areas. **a** Preserved optic tract (arrow) and severe degeneration of the other regions. **b** Preserved lateral geniculate body (arrow) and severe degeneration of other regions. (Patient 7, Bar = 5 mm, Klüver–Barrera staining)

The optic tract and optic radiation were well preserved in all the patients (Fig. 4). The lateral geniculate body was examined in nine patients, of which eight showed comparative preservation. In the striate cortex, although there were a few NCI in six patients (patients 1–4 with pTDP-43-ir NCI, and patients 7 and 8 with FUS-ir NCI), mild neuronal loss and gliosis were observed only in patient 4. The nucleus basalis of Meynert was examined in eight patients, showing no neuronal loss although a few NCI were found in patients 4, 6 with pTDP-43-ir NCI and in patient 11 with SOD1-ir NCI.

#### Cerebral cortical findings by accumulated protein

**Fig. 5:**
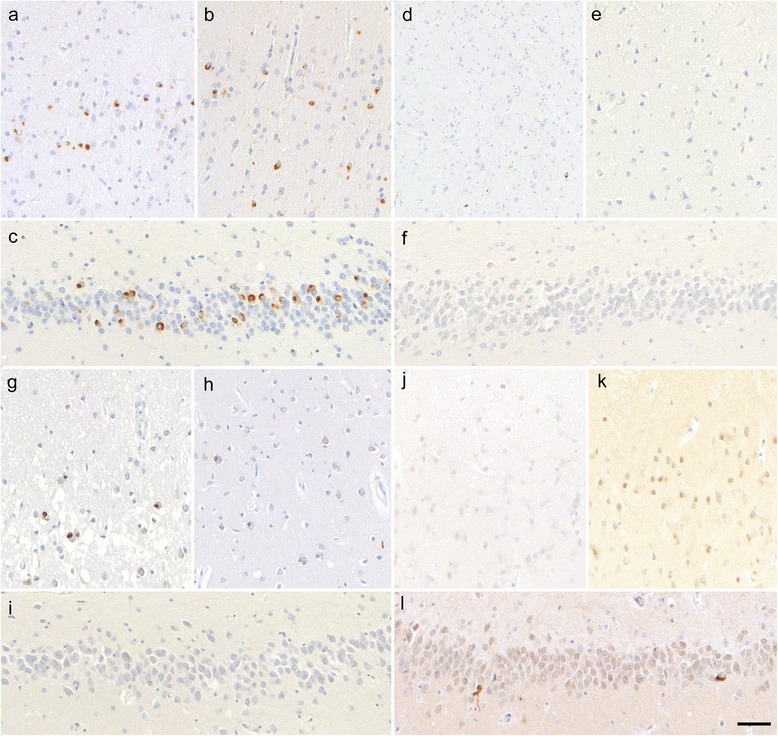
Immunohistochemical features of cerebral lesions. **a**–**c** Patient 2 with phosphorylated TAR DNA-binding protein 43 (pTDP-43)-immunoreactive (ir) neuronal cytoplasmic inclusions (NCI) in the hippocampal dentate granule neurons (**c**) had occasional NCI in the frontal (**a**) and temporal (**b**) cortex. **d**–**f** Patient 6 without pTDP-43-ir NCI in the hippocampal dentate granule neurons (**f**) had rare NCIs in the frontal cortex (**d**) and no NCIs in the temporal cortex (**e**). **g**–**i** Patient 7 with occasional fused in sarcoma (FUS)-ir NCI in the frontal cortex (**g**) and rare NCI in the temporal cortex (**h**) had no NCI in the hippocampal dentate granule neurons. **j**–**l** Patient 8 without FUS-ir NCI in the frontal (**j**) and temporal cortex (**k**) had rare NCI in the hippocampal dentate granule neurons (**l**). Bar = 50 μm

Among six patients with pTDP-43-ir NCI (patients 1–6), four (patients 1–4) had NCI in the hippocampal dentate granule neurons and showed neuronal loss and gliosis with NCI in the hippocampal subiculum, and the frontal and temporal cortex (Fig. 5a–c). Patients 5 and 6, lacking NCI in the dentate granule neurons in the hippocampus, demonstrated the occurrence of NCI, limited to the frontal cortex, in the absence of neuronal loss (Fig. 5d–f). The pTDP-43-ir pathological pattern of all six patients was consistent with type B [12].

One (patient 7) with FUS-ir NCI showed frontal and temporal cortical degeneration with compacted occurrence of smaller NCI in the absence of NCI in the hippocampal dentate granule neurons (Fig. 5g–i). The other (patient 8) had noncompact NCI in the hippocampal dentate granule neurons, and their cerebral cortex was preserved with rare NCI (Fig. 5j–l).

The cerebral cortex, with the exception of the motor cortex, of all three patients with SOD1-ir NCI was preserved. A few NCI were scattered in the temporal cortex only in patient 9.

## Discussion

As noted in our previous reports [9, 19], patients with ALS that progressed to Stage V showed rapid clinical deterioration, and tended to need TIV within 24 months of ALS onset. The onset of lesions varied, and the onset lesions and the time from onset to resulting in Stage V did not appear to be related. Subsequently, their condition progressed to Stage V due to complete ophthalmoplegia in addition to total quadriplegia. This clinical course is similar to that of the previously reported patients who progressed to Stage V (Table 3) [11, 14, 26, 28]. However, a few patients required TIV over 24 months after the onset, such as our patient 3 with pTDP-43-ir NCI, and three patients reported in the existing literature, showing pTDP-43-ir NCI [11], basophilic inclusions [14], and a mutation in *OPTN* [28], respectively (Tables 1 and 3). These patients showed that patients who required TIV after more than 24 months from disease onset could not necessarily avoid progression to Stage V.

**Table 3:** Clinicopathological characteristics of previously reported patients in communication Stage V

			Time from onset to clinical event, months													
Sex	Age at onset (years)	Disease duration (months)	Need for tracheostomy invasive ventilation	Progression to total quadriplegia	Development of overt oculomotor limitation	resulting in communication Stage V	Communication Stage V to death(months)	Accumulated protein	Neuropathology Brain weight	Upper and lower motor neurons	Substantia nigra	Globus pallidus	Subthalamic nucleus	Brainstem reticular formation	Cerebellar efferent system	Reference
M	38	60	11	20	20	32	28	ubiquitin	1300	+++	+++	+++	na	+++	++	[14] Patient 1
M	38	156	60	132	96	144	12	ubiquitin^a^	1230	+++	+++	na	na	++	+++	[14] Patient 2
M	63	144	36	na	36	na	na	TDP-43	830	+++	+++	+	na	+++	+	[11]
F	33	288	24	na	na	156	84	a mutation in *OPTN*		N/A	N/A	N/A	N/A	N/A	N/A	[28] Case 1^b^
M	35	240	36 ≥	na	na	36	204	a mutation in *OPTN*		N/A	N/A	N/A	N/A	N/A	N/A	[28] Case 2^b^
M	44	27	6	15	15	26	1	na	1320	+++	+	+	+	+++	+	[26]

*M* male, *F* female, *na* not available, *N/A* not applicable, *OPTN* the optineurin gene

^a^ubiquitin-immunoreactive basophilic inclusions

^b^Clinical report

Neuropathologically, regardless of the type of accumulated proteins, all the patients shared the feature of severe degeneration in the substantia nigra, globus pallidus, subthalamic nucleus, brainstem reticular formation, and mild to severe degeneration of the cerebellar efferent system, in addition to the severe degeneration in both upper and lower motor neurons. It is likely that the constellation of such lesions is common in patients with ALS who progress to Stage V, because the patients reported in the literature who had progressed to Stage V also demonstrated a similar constellation (Table 3) [11, 14, 26]. In addition, Miki et al. [13] reported a patient with ALS who required TIV and did not progress to Stage V, and discussed this with regard to pallido–nigro–luysian degeneration. They concluded that the pallido–nigro–luysian degeneration may be involved in the ALS disease process [13]. Another patient with ALS who required TIV and did not progress to Stage V also showed pallido–nigro–luysian degeneration [23]. These two patients who did not progress to Stage V showed no apparent degeneration in the brainstem reticular formation or cerebellar efferent system, although they did show pallido-nigro-luysian degeneration similar to the patients who progressed to Stage V [13, 23]. Therefore, it is likely that the degeneration of the brainstem reticular formation and the cerebellar efferent system in addition to degeneration of the pallido–nigro–luysian system is necessary for progression to Stage V.

Relatively mild degeneration of the cerebellar efferent system was seen in patients who died shortly after progressing to Stage V (patients 3, 5, 6, 8, 11) and has been reported in one patient [26]. The time for progression from Stage V to death may alter the degree of degeneration in the cerebellar efferent system.

**Fig. 6:**
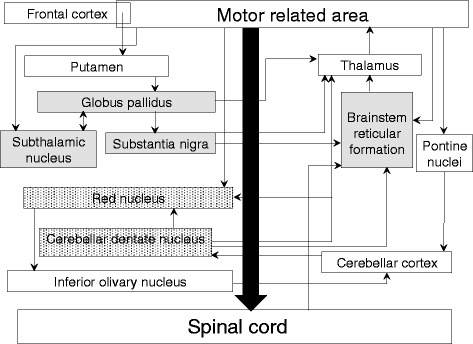
The connections of motor neuron-related area. The common lesion of the ALS patients who progressed to Stage V is emphasized. All the common lesions related to the motor neurons and pyramidal tract. Shaded areas: lesions that showed moderate or severe degeneration in all the patients regardless of the time for progression to Stage V to death or type of accumulated protein. Stippled areas: lesions that showed a different degree of degeneration depending on the time from progression to Stage V to death

Neuroanatomically, the common lesions of patients with ALS who progressed to Stage V appeared to be related to each other through connections with the motor-related area (Fig. 6) [4]. The degeneration of motor neurons, which are the basic lesions in ALS, is so severe in patients who progress to Stage V that the degeneration of associated areas appeared to occur secondarily due to degeneration of motor neurons. We could not evaluate the precise relationship between neuronal loss and occurrence of NCI because of the severe neuronal loss. Brettschneider et al. described a pTDP-43 staging algorithm, and presented evidence for a possible sequential dissemination of pTDP-43 pathology [2, 3]. However, applying the stages of pTDP-43 to the present patients with pTDP-43-ir NCI, the inferior olivary nucleus, which was mentioned as an early lesion [2, 3], showed relatively mild neuronal loss even in communication Stage V, and the subthalamic nucleus, which was mentioned as a lesion having no NCI until late stages [2, 3], showed moderate to severe degeneration in communication Stage V. At least in the patients with pTDP-43-ir NCI, we speculate that the ease of occurrence of NCI and the speed of degeneration in the patients with ALS may differ depending on each lesion, as follows: (1) Lesions that showed a matched degree of appearance of NCI and neuronal loss, such as in the motor neurons, globus pallidus, and substantia nigra, (2) lesions that had NCI from an early disease phase without rapid neuronal loss, such as in the inferior olivary nucleus, (3) lesions although having rare NCI in early disease phases, subsequently showed severe neuronal loss, such as in the subthalamic nucleus, and (4) lesions that had rare NCI without obvious neuronal loss, such as in the lateral geniculate body and striate cortex in the visual pathway, which were preserved from lesions in patients who progressed to Stage V [22].

In this study, patient 2 with pTDP-43-ir NCI and patient 10 with SOD1-ir NCI [10] had inferior olivary hypertrophy. It is noteworthy that inferior olivary hypertrophy can occurs in ALS, regardless of accumulated protein, likely to progressive supranuclear palsy [6], Creutzfeldt–Jakob disease [15], or cerebrovascular disease [5].

A distinct pattern of protein accumulation was found in the cerebral cortex. First, in patients with pTDP-43-ir NCI (Table 2, Fig. 5a–f), patients with no NCI in the hippocampal dentate granule neurons (patients 5, 6) showed limited degeneration in the frontal cortex, and had relatively preserved brain weight. By contrast, patients with NCI in the hippocampal dentate granule neurons (patients 1–4) showed marked degeneration in the temporal cortex and hippocampal subiculum; and NCI were observed in the frontal, temporal, and occipital cortex. The characteristics of the cerebral cortical features in the present patients could be classified according to the presence of NCI in the hippocampal dentate granule neurons as described by Nishihira et al. [21]. Moreover, we identified that the patient with a Nishihira’s Type 1 distribution pattern also progressed to Stage V. Interestingly, the relationship between NCI in the hippocampal dentate granule neurons and cerebral cortical degeneration showed a contrasting trend in patients with pTDP-43-ir NCI and FUS-ir NCI in this study as follows: patient 7 [17], who lacked NCI in the hippocampal dentate granule neurons showed severe atrophy of the cerebrum, whereas patient 8 [16], who had NCI, showed no atrophy of the cerebrum (Table 2, Fig. 5g–l). Second, the cerebral cortex of all 3 patients with SOD1-ir NCI was preserved with rare NCI. Patients with a mutation in *SOD1* were reported as having frontotemporal dementia (FTD) and decline of cognitive function was rare [25, 29]. Only one patient with a mutation in *SOD1* showing frontotemporal lobar degeneration (FTLD) underwent a detailed clinicopathological analysis [18]. Taking these findings together, it is likely that the cerebrum of patients with SOD1-ir NCI may be preserved even in the patients who progressed to Stage V. That there were three patients who showed a preserved cerebrum (patients 9–11), and that cerebral lesions shared by all patients and severe neuronal loss were missing in the present patients, suggest that cerebral cortical involvement was not necessary to progress to Stage V.

It is important to differentiate the present patients from patients showing FTLD. There is a limitation in that the decline of cognitive function cannot be assessed in the patients who progressed to Stage V. However, none of the patients had any clinical manifestation of cognitive or behavioral impairment while they could communicate, so they were not diagnosed as having FTD [25]. Furthermore, it was revealed that the patients who progressed to Stage V did not always show pathological characteristics of FTLD.

## Conclusion

In the present study, we evaluated only a small number of patients because the patients who progressed to Stage V were limited to 3.4 % of all patients with ALS who underwent autopsy. Nevertheless, we identified that the patients who progressed to Stage V, including the patients reported previously, had common lesions in the pallido–nigro–luysian system, brainstem reticular formation, cerebellar dentate nucleus, superior cerebellar peduncle, and red nucleus, besides motor neurons. Moreover, we clarified specific characteristics in the relationship between accumulated protein and the cerebral cortical lesions. Thus, there may be common lesions that can identify patients with ALS who progress to Stage V. We consider that attention to the occurrence of symptoms of the extrapyramidal motor system and nonmotor system, and a radiological assessment of common lesions are important for predicting communication disability, especially in the patients who progressed rapidly from disease onset to TIV.
